# Re-imagining the future of diagnosis of Neglected Tropical Diseases

**DOI:** 10.1016/j.csbj.2017.02.003

**Published:** 2017-02-21

**Authors:** Rosanna W. Peeling, Debrah I. Boeras, John Nkengasong

**Affiliations:** a LONDON School of Hygiene and Tropical Medicine, United Kingdom; b Centers for Disease Control and Prevention, USA

**Keywords:** Neglected tropical diseases, Diagnostics, Control, Elimination, Surveillance

## Abstract

Neglected Tropical Diseases (NTDs) affect an estimated 1 billion people in 149 countries. The World Health Organization (WHO) prioritised 17 NTDs for control and elimination by 2020 and defined a Road Map to help countries reach these goals. Improved diagnostics for NTDs are essential for guiding treatment strategies at different thresholds of control, interruption of transmission, elimination and post-elimination surveillance. While substantial progress has been made in the last decade with chemotherapy, the same cannot be said of diagnostics, largely due to the perceived lack of a commercially viable market for NTD diagnostics.

New sample in-answer out nucleic acid amplification technologies that can be performed at the point-of-care offer improved performance over current technologies and the potential to test for multiple pathogens using a single specimen. Finding commonalities for different NTDs in terms of geographic overlap, sentinel populations and treatment strategy will allow NTD programs to leverage these innovations to build cost-effective multiplex surveillance platforms. Connectivity solutions linking data from diagnostic laboratories and POC test readers/devices provide opportunities for automated surveillance systems to make health systems more efficient, improving patient outcomes and assessing impact of interventions in real time. New models of public–private product development partnerships are critical in leveraging diagnostic innovation in other priority area for better diagnosis, control and elimination of NTDs.

## 1 Introduction

Neglected Tropical Diseases (NTDs) affect an estimated 1 billion people in 149 countries in the developing world and give rise to a myriad of adverse impacts, including anemia, blindness, cognitive impairment, and death [1], [2]. The World Health Organization (WHO) prioritised 17 NTDs (Buruli ulcer, Chagas disease, cysticercosis, Dengue, drancunculiasis (Guinea worm disease), echinococcosis, endemic treponematoses (Yaws), foodborne trematode infections, human African trypanosomiasis (HAT), visceral leishmaniasis (VL), leprosy, lymphatic filariasis (LF), onchocerciasis, rabies, schistosomiasis, soil-transmitted helminthiases (STH), and trachoma) for control and elimination. These NTDs were selected on the basis that their transmission characteristics or treatment opportunities make them good candidates for control and elimination [3].

WHO, the Bill & Melinda Gates Foundation and 13 leading pharmaceutical companies, at a meeting in London in 2010, resolved to sustain, expand and extend programmes to ensure the necessary supply of drugs and other interventions to eradicate Guinea worm disease, eliminate LF, leprosy, HAT and blinding trachoma; and to control schistosomiasis, STH, Chagas disease, VL and onchocerciasis by 2020 [4]. The London Declaration ensured the donation of drugs for NTDs but diagnostics, critically needed for monitoring progress towards elimination and assessing the impact of special intervention, was not included in the Declaration as a priority.

In 2013, WHO defined a Road Map including five key interventions to help countries reach the 2020 goals [3]. These are: (i) preventive chemotherapy based on large-scale use of safe, single-dose medicines at regular intervals (i.e. mass drug administration, MDA); (ii) innovative and intensive case management; (iii) vector ecology and management; (iv) improvements in water, sanitation and hygiene in NTD-endemic areas; and (v) veterinary interventions to protect and improve human health [2], [3]. While substantial progress has been made in the last decade with chemotherapy reaching a billion people in 2014 [5], the same cannot be said of diagnostics needed to guide chemotherapy and for surveillance, largely due to the perceived lack of a commercially viable market for NTD diagnostics.

Diagnostics are needed, with the exception of Guinea worm which is associated with unmistakable clinical features, to monitor and certify elimination of NTDs. Dowdle advocated for ‘practical diagnostic tools of sufficient sensitivity and specificity to detect levels of infection that can lead to transmission’ as an essential requirement for disease elimination or eradication [6]. The lack of a clear diagnostic strategy has resulted in limited surveillance data, with countries often using only using disease burden as a proxy for countrywide data. Solomon et al. suggested that country programs for control and elimination of NTDs demand improved diagnostic tools in order to “guide decisions on the required intensity, frequency, and duration of intervention and to conduct surveillance for re-emergency of infection after elimination” [7].

This article examines recent advances in diagnostic technologies driven by high profile, high burden diseases such as HIV and tuberculosis, emerging global health threats such as antimicrobial resistance and emergencies such as outbreaks of SARS, Ebola virus disease and Zika virus infections, and determines how these innovations can be leveraged to improve the diagnosis and surveillance of NTDs.

## 2 Investments in diagnostic innovation

Accurate diagnostic tests are commercially available for most infectious diseases. These tests are laboratory based and hence not widely accessible in the developing world, where laboratory infra-structure is often limited. The need for increasing access to diagnostics for patients with HIV and TB in the development world has led to major investments in more accessible diagnostic technologies that can be used at the point-of-care within the last decade.

### 2.1 Diagnostic innovations for HIV and tuberculosis

UNAIDS set 90–90–90 targets for HIV which call for 90% of those who are infected know their HIV status, 90% of those infected be put on treatment, and 90% of those on treatment achieve viral suppression [8]. Globally, countries are now at 50%, 37% and 25% respectively with regard to these targets [9]. To realize these targets, good performing diagnostic and monitoring tests that are widely accessible for populations in remote areas or marginalized from care are needed.

The diagnosis of TB traditionally depends on smear microscopy and chest radiography, both of which are insufficiently sensitive and specific. Culture is highly specific but requires up to 6 weeks for a result. Molecular methods are now used in developed countries, but the cost and requirement for laboratory infrastructure and skilled technical staff prevent their adoption in low-resource settings. Modeling studies show that if a test of 85% sensitivity and 97% specificity can be performed during a single patient visit and is widely implemented, 625,000 lives could be saved each year [10], [11].

Investments in point-of-care (POC) technologies have resulted in a range of highly sensitive and specific HIV and TB molecular assays that can be performed in remote settings [12]. These tests are designed in a “sample-in answer-out” format, requiring minimal training, and providing a result in 1–2 h. Molecular platforms which can detect *Mycobacterium tuberculosis* and rifampicin resistance with high accuracy in just under 2 h have transformed TB diagnosis in both the developed and developing world. POC tests for CD4 enumeration have been commercially available for a few years now and have shortened delay in HIV treatment [13]. POC assays for HIV early infant diagnosis and viral load are currently under evaluation and will be implemented shortly. The expectations are that these tests will increase access to HIV viral load monitoring and early infant diagnosis with faster turn-around time for results to patients, increased linkage to care and reduced loss-to-follow up, and decreased risk of drug resistance.

### 2.2 Diagnostics for global health emergencies

Further investments in technological innovation are being driven by successive global health emergencies including SARS, flu, MERS CoV, Ebola virus disease and Zika virus infection. WHO calls for development of open platform technologies to accelerate the development, validation and production of vaccines, therapeutics, diagnostics and reagents for infectious diseases of epidemic potential as part of the WHO Blue Print for R&D preparedness [14]. At the 2015 G7 Ministers of Health meeting, there was consensus on “…continued financing, collaboration and coordination … through initiatives such as the WHO blueprint for R&D preparedness and the Global Research Collaboration for Infectious Disease Preparedness (GloPID-R)” [15].

The priority pathogens for the WHO Blue Print R&D include Crimean Congo Haemorrhagic Fever (CCHF), Filoviruses, Lassa, Severe Coronaviruses such as the Corona viruses that caused SARS and the Middle East Respiratory Syndrome (MERS CoV), Nipah virus, Rift Valley fever, Chikungunya, Zika and Severe fever with Thrombocytopenia Syndrome (SFTS), an emerging tick-borne infectious disease caused by the SFTS virus (SFTSV), a novel and highly pathogenic phlebovirus in the family Bunyaviridae. As a result of the call for open platform technologies, industry and public sector developers have initiated collaborations to work together to accelerate product development.

These open platform technologies are being developed by companies that have already developed highly accurate POC tests for HIV and TB. Many of these companies have also developed cartridges to detect Ebola and Zika viruses using the same platform. Some companies have already developed a broad menu of infectious diseases on the same platform. Advocacy and incentives, such as advance market commitment, would be needed for these companies to apply their technologies to NTDs. A review of emerging molecular technologies that have been developed and can be used in resource-limited settings has been published [16].

### 2.3 Antimicrobial resistance

Antimicrobial resistance (AMR) is one of the greatest public health challenges of this century with an estimated 25,000 deaths and over €1.5 billion a year in healthcare expenses and productivity losses in Europe alone [17], [18], [19]. In the United Kingdom AMR Strategy, two of the five AMR targets require the development of diagnostics that will rapidly identify infections that require antibiotics and of assays that can be used to identify and track patterns of antimicrobial resistance [18]. Rapid POC tests can also be used in drug trials to reduce the cost and length of trials, as target populations can be identified and recruited without expensive laboratory tests and procedures. Diagnostic technologies for AMR currently available and in the pipeline are described in a compendium prepared by the Oxford Centre for Evidence-based Medicine [20].

The challenge is to create a cost-effective, accurate, rapid, and easy-to-use test for bacterial infections that will allow health professionals worldwide to administer the right antibiotics at the right time or rule out antibiotic use by identifying viral infections. A number of initiatives to incentivise public and private sectors to develop rapid diagnostic tests and assays for AMR surveillance have been announced. The £10 million Longitude Prize is a prize fund for a diagnostic tool that can be used to rule out antibiotic use or help identify an effective antibiotic to treat a patient [21]. The Horizon 2020 prize of €1 million put forth by the European Commission is to incentivise better use of antibiotics for respiratory infections through the development of a rapid test that will allow healthcare providers to distinguish, at the point of care, between patients with respiratory tract infections that require antibiotics and those who can be managed safely without antibiotics [22]. The US National Institutes of Health has also offered a prize of up to $20 million to the first group(s) to develop a rapid, POC diagnostic test to be used by health care providers to identify highly resistant bacterial infections to promote responsible use of antibiotics [23].

Promising high throughput array technologies for pathogen detection coupled with detection of antimicrobial susceptibility patterns for AMR surveillance will be needed to guide patient management and improve our understanding of the emergence and spread of resistance. How can the NTD community leverage these investments in diagnostic innovation to improve patient management, disease control and surveillance towards the elimination of NTDs?

## 3 Innovation in diagnostics for NTDs

It is neither feasible nor efficient for countries to manage 17 NTDs as individual vertical programmes, each with its own range of diagnostic and surveillance tools. There is a need to find commonalities and develop a unifying strategy for the diagnosis and surveillance of NTDs. Commonalities that can be considered are:

### 3.1 NTDs with a common control strategy such as mass drug administration

Different types of diagnostics are needed to inform policy decisions at different stages of control for NTDs for which MDA is the main control strategy [24]. After multiple rounds of MDA, highly sensitive and specific diagnostics are needed to locate “hot spots” of residual infection. For schistosomiasis, the intensity of transmission decreases with decreasing prevalence of infection. Microscopy is no longer sufficiently sensitive to detect residual infection in communities when the number of eggs per gram of stool falls below 40. Antigen or nucleic acid amplified tests (NAATs) are needed to replace microscopy to identify communities in which transmission is still occurring. However, most antigen detection tests using enzyme immunoassays or lateral flow tests have not been shown to be sufficiently sensitive and current NAATs are too expensive and technical demanding for community based screening. As NAATs become available in POC format and have data transmission capabilities, these devices can be taken around to different communities to determine the stage of control and to inform treatment strategies.

### 3.2 Overlapping geographic endemicity and common sentinel populations

Much progress has been made in mapping the geographical distribution of different NTDs. In areas with overlapping NTDs, if the sentinel population is similar, there could be cost saving and efficiencies in collecting a single specimen for surveillance of multiple NTDs. For LF, trachoma, schistosomiasis, onchocerciasis, the sentinel population is children under ten years of age. Multiplex antibody detection assays that can be performed on a single dried blood spot from finger pricked blood offer the most cost-effective means of surveillance [7].

### 3.3 Common treatment

Both Yaws and trachoma require community based treatment with one or more rounds of mass treatment with azithromycin. Although the goals of the yaws and trachoma programs are different, and the implementation of interventions may not be fully aligned, there may be potential synergies between the two programs in the use of duplex diagnostic tests for case finding and surveillance [25].

### 3.4 Advances in diagnosis of viral infections

The diagnosis of viral infections is traditionally made by demonstrating a seroconversion to IgM or a 4-fold rise in IgG titers between acute and convalescent sera taken more than 10 days apart. Such paired sera are difficult to obtain. Hunsperger et al. showed recently that > 90% of dengue cases can be diagnosed from a single serum specimen collected during the first 10 days of illness by testing it with either DENV-RT-PCR + IgM ELISA or NS1 antigen ELISA + IgM ELISA [26]. This opens up an exciting possibility of using test combinations to diagnose viral NTDs with a single blood sample taken during the acute phase of infection. It offers a tremendous improvement over our current struggles with global health emergencies most of which are caused by outbreaks of viral diseases.

## 4 The promise of connectivity to strengthen the efficiency of health systems

In 2001 Heymann and Rodier drew our attention to the capability of ‘infectious disease intelligence’ to improve early warning capacity for potential worldwide public health problems and possibly diminish or even prevent the spread of infectious diseases [27]. Connectivity solutions for testing at the point-of-care can now be used to provide timely information on testing, trends, quality assurance and can be coupled to optimize supply chain management. Alerts can be built into connectivity systems to support disease surveillance and outbreak investigations.

Nowadays there are a lot of POC devices that can digitize data and transmit them to a central database [28]. This integration of digital technology into diagnostics will allow the ministry of health to have up-to-the-hour information on what is happening across the country. Connectivity solutions can also be used to link in proficiency testing results from each testing site and alerts for remedial action with the ultimate goal of improving the accuracy of testing across the country. Supply chain management can be informed through links to the testing database, which is another opportunity to strengthen the healthcare system.

In some countries, bi-directional connectivity not only allows healthcare sites to send information to the central database, but the database can report trends back to the site operators. Information about the devices can also be tracked, such as frequency of device failure and error rates. More research is needed to understand middleware solutions as to whether they could extract the same set of data from different data clouds, what kind of IT knowledge would be needed to manage such systems in the field and what advice countries would need about human resources and training. This would all make the health system a lot more efficient and the information would be very useful for designing control programs, tracking disease trends and assessing the impact of interventions in real time.

## 5 The challenges of implementing POC diagnostics

While POC tests can offer rapid identification of the causes of infections and enable appropriate prescribing, taking testing outside of laboratories can add stresses to a weak or fragile health care system. Leadership and infrastructure for critical decision making on the adoption of new diagnostic technologies are often fragmented or absent in many countries. Companies with novel diagnostic technologies often face long delays in regulatory approval and unnecessary expenditure associated with excessive duplication of clinical trials in countries they intend to market. Unlike the regulation and adoption of new drugs and vaccines, global governance and oversight to ensure quality and efficiency are lacking in diagnostics.

The barriers faced in implementing testing at the point-of-care are often not technological, but constraints inherent in the health care system. To implement a successful POC diagnostics programme, the engagement of policy makers, stakeholders and partners to ensure buy-in and align necessary resources is a critical first step. Training and certification of vast number of health care providers to use POC diagnostics, and ensuring an effective supply chain for tests and drugs across the country present an enormous challenge to health systems that are already suffering from a critical shortage of human resources. Quality assurance systems, that include providing proficiency testing, sites visits, and corrective action, are essential to ensure that accurate results are being used to guide treatment decisions, and to ensure that control strategies are based on accurate surveillance data. Studies have shown that deployment of POC diagnostics can be an opportunity to improve health outcomes and strengthen health systems [29], [30], [31]. Overcoming challenges will in turn motivate health care workers and increase capacity and efficiency to test and treat appropriately. RDT introduction and implementation must be culturally sensitive and accompanied by an inter-connected system to monitor the necessary processes. Another key barrier to the appropriate use of POC diagnostics is the need to train clinicians to create a demand for them and then use the test results to effectively manage the patients. Improving the laboratory-clinician interface is an important but often neglected aspect of testing introduction.

## 6 The way forward

Appropriate diagnostics to monitor disease trends and assess the impact of interventions are essential for guiding treatment strategies for NTDs at different thresholds of control, interruption of transmission, elimination and post-elimination surveillance.

New sample in-answer out nucleic acid amplification technologies offer improved performance over antigen detection POCTs and the potential to test for multiple pathogens using a single specimen. Recent technical advances have led to improved diagnostic tests for NTDs, which are sensitive, specific, able to diagnose multiple infections using a single specimen, and can be used at the point of care. Since there is extensive geographical overlap between different NTDs targeted by MDA, multiplex surveillance platforms are likely to prove cost-effective.

Rapid deployment of these new technologies for the control and elimination of NTDs can present multiple challenges on fragile health systems, including quality assurance and stockout of supplies needed for testing and treatment. Connectivity solutions linking data from diagnostic laboratories and POC test readers/devices provide opportunities for automated surveillance systems to make health systems more efficient, improving patient outcomes and assessing impact of interventions in real time. New models of public–private product development partnerships are critical in leveraging diagnostic innovation in other priority area for better diagnosis, control and elimination of NTDs.
